# Factors that facilitate treatment uptake for women with alcohol use disorders in high-income countries: A systematic review

**DOI:** 10.1177/17455057251363713

**Published:** 2025-08-27

**Authors:** Melise Ammit, Jo River, Mark Montebello, Angela Dawson

**Affiliations:** 1 University of Technology Sydney, Faculty of Health, Ultimo, NSW, Australia; 2 Northern Sydney Local Health District, St Leonards, NSW, Australia; 3 University of Sydney, Camperdown, NSW, Australia

**Keywords:** alcohol-related disorders, alcoholism, women, females, facilitators, treatment seeking, enablers

## Abstract

**Background:**

Harmful alcohol use among some groups of women is increasing globally. Despite being susceptible to negative health impacts, women are less likely than men to seek treatment and can face barriers of access, acceptability, and affordability to treatment.

**Objectives:**

This study aims to identify key factors affecting treatment access for women with alcohol use disorders (AUDs), and to identify individual, social, and organizational factors that facilitate treatment uptake.

**Design:**

Systematic review with narrative synthesis, guided by a social-ecological framework to identify organizational, individual, and societal enablers of treatment uptake among women with AUDs.

**Methods:**

Six electronic databases were systematically searched for studies published between 2000 and 2024 in high-income countries. Data on study design, sample characteristics, interventions, and outcomes were extracted and synthesized.

**Results:**

Twenty-five studies, conducted in various countries, identified factors affecting women’s treatment uptake. Key themes included individual motivators linked to problem perception, alcohol use severity, age, relationships, and family cohesion; societal motivators influenced by social norms and relationships; and organizational themes of accessibility, acceptability, and affordability were positively affected by healthcare provider knowledge of treatment options.

**Conclusion:**

Facilitating factors of treatment uptake for women with AUDs include relational and personal goals, societal environments, normative expectations, and the knowledge of alcohol treatment from individual and organizational perspectives. Sober curiosity movements, alcohol-free months, and digital strategies can positively impact women’s alcohol treatment uptake.

## Plain language summary

What enables women with problematic alcohol use to access treatment in high-income countries: A review

Harmful alcohol use among some groups of women is rising globally, but women are less likely than men to seek treatment, despite the health risks. Research suggests that more focus is needed on how to help women access treatment for alcohol use issues. This study reviewed research from high-income countries in the past 20 years to understand what factors help women with alcohol use disorders get treatment. The review included 25 studies that looked at individual, societal, and organizational influences on treatment access. The key findings identified that personal factors, such as how serious women think their drinking problem is, their relationships, and family support, play a role in whether they seek treatment. Social factors, like societal norms and relationships, also influence treatment decisions. Organizational factors, like how available and affordable treatment is, along with healthcare professionals’ knowledge about treatment options, also make a difference. The review concluded that making treatment easier for women involves understanding their personal goals and social environments, alongside the broader healthcare system. Movements like “sober curiosity” and alcohol-free months, as well as digital healthcare innovations following COVID-19, might help encourage more women to seek alcohol treatment in the future.

## Background

Alcohol use disorders (AUDs) are characterized by impaired control of alcohol consumption, compulsion to drink, and withdrawal symptoms upon cessation.^1^ The harmful use of alcohol is a causal factor in more than 200 disease and injury conditions and accounts for 5.1% of the worldwide burden of disease. AUDs are more prevalent in high-income countries than in low- and lower-middle-income countries and Muslim-majority countries.^2^ In high-income countries, population rates of alcohol consumption at harmful levels vary from approximately 19% in the United States, 31% in Australia, 28% in England and Scotland, and 15% of people in Ireland meet the criteria for AUD.^3–7^

Despite the availability of effective treatments for AUD treatment-seeking is low.^8,9^ Attitudinal barriers exist, with many individuals believing the problem is not severe enough to warrant professional intervention or will be resolved on its own. The reliance on personal willpower reflects widespread misconceptions about AUD and its treatment.^9^ Although factors such as psychiatric comorbidities and severity of alcohol-related problems predict higher treatment uptake, barriers such as stigma and a lack of perceived need for care, continue to hinder treatment access.^10–12^ Women are less likely to seek treatment than men and the gap between need and receipt of treatment is larger for women than for men, with an average delay of 10 years between recognizing the problem and accessing help.^13^

Over the past decade, female harmful alcohol use has been an emerging trend in many countries. The World Health Organization reports that the absolute global number of currently drinking women has increased and is predicted to continue increasing, especially in North and South America.^14^ In Australia, women aged 45–60 who consume more than two standard drinks per day has increased from 8.8% in 2001 to 11.7% in 2019,^15^ and women aged 30 to 39 have increased single-occasion risky drinking from 17% in 2001 to 21% in 2016.^16^ Similarly in 2021, 9.5% of women in the United States were reported to have an AUD.^17^ One in every 30 women aged 16 or older in the United Kingdom, have an AUD,^18^ and the overall rate of hazardous drinking among women aged 34–70 in New Zealand is approximately 21%.^19^

The size of the gender gap in alcohol use differs between countries and their cultures. For example, in Australia, among women aged 39 to 59, parity with men’s consumption of alcohol has almost been reached and in the United States drinking patterns of women and men are almost comparable.^20,21^ A similar trend has occurred in New Zealand and Norway, whereas in India, male use of alcohol outweighs women by 12:1 suggesting that culturally prescribed gender roles shape drinking behavior.^22^

Women are more vulnerable than men to the physical effects of alcohol due to differences in body weight and metabolism and are more susceptible to liver disease.^23^ Additionally, variations in stress, immune system functioning, and hormonal factors between the sexes alter how alcohol impacts the immune system, leading to a quicker onset of alcohol-related health problems in women.^24^ Furthermore, women experience more rapid and severe alcohol-related health consequences than men – a phenomenon known as the telescoping effect – which is more prominent in alcohol use rather than other drug use.^25^ Gender differences in alcohol use trajectories highlight that women who start drinking regularly before the age of 18 are more likely to progress from no problems to severe problems without showing the same recovery patterns as men.^26^

Women with moderate intake (15–30 g/day) have a 10% higher risk ratio for mortality from cardiovascular disease possibly due to higher blood ethanol levels and the risk of liver dysfunction that contributes to morbidity.^27^ Furthermore, alcohol is a risk factor for the incidence of breast cancer; as little as one extra standard drink a day can increase the risk of breast cancer by 5% for premenopausal women and 9% for postmenopausal women.^28^

Hormonal differences between men and women can affect alcohol use, as menstrual-cycle stage and estrogen levels can influence “drug-liking” and craving.^29^ A systematic review by Salari et al.^30^ reported a significant association between alcohol intake and sexual dysfunction in women, with 55% of study respondents reporting lower libido, and 52% in difficulty reaching orgasm.^30^ In addition, menopause-related stress and depression can affect alcohol consumption and result in alcohol-related disease and injury, such as falls, stroke, and osteoporosis during the menopausal years.^31^ And, while moderate alcohol consumption, defined as less than 12.5 g/day (about 1 standard drink), was associated with a lower risk of dementia, excessive drinking (more than 23 standard drinks per week) was associated with a higher risk of alcohol-related cognitive impairment.^32^

Women are more likely than men to experience childhood trauma, increasing their risk of using alcohol to cope with emotional distress. They also report higher rates of co-occurring psychiatric conditions, such as mood disorders, personality disorders, and post-traumatic stress disorders (PTSD), which can contribute to AUD development.^33^ Furthermore, trauma-exposed individuals, particularly women, often use alcohol to manage PTSD, increasing their vulnerability to revictimization, and perpetuating the cycle of trauma.^34^

Alcohol use has historically been seen as a male-dominated behavior, and treatment approaches and settings can reflect this view.^35^ Women can experience barriers to treatment including a lack of recognition that their alcohol use is a problem, previous negative experiences with alcohol treatment, or lack of knowledge of treatment availability and efficacy.^36^ Research indicates that women can experience greater shame about alcohol use compared to men.^37,38^ Affordability and accessibility factors, fears regarding the impact on employment and children, and childcare availability, also pose significant barriers to alcohol treatment for women.^39,40^ Moreover, some women in a primary caregiving roles may avoid seeking treatment due to fears about mandated reporting laws and child removal – which can be further exacerbated by healthcare providers’ lack of education on substance use treatment and explanation of mandated reporting laws.^41,42^

Although systematic reviews exist that focus on specific populations or interventions for women,^43–49^ none focus on the factors facilitating treatment uptake. Given the potential gender disparities that exist in accessibility to AUD treatment, this study focused on identifying key factors affecting women’s treatment access, which is a necessary first step to identifying ways to improve treatment access and acceptability for women.

## Method

This systematic review sought to determine factors that facilitate treatment access for women with an AUD in high-income countries as defined by the World Bank. We employed a narrative synthesis and applied a social-ecological framework – based on the model first introduced by Bronfenbrenner in the 1970s – as it examines human behavior within interconnected personal, relational, healthcare, societal, and economic layers, providing a nuanced understanding of the factors influencing women’s decisions to seek treatment.^50^

### Search strategy

The search strategy was developed in consultation with a research librarian. Examples of the search terms used were: “alcohol dependence,” “alcoholism,” “alcohol use disorders,” “alcohol-related disorders,” “women,” “female,” “treatment-seeking,” “facilitators,” “enablers,” “barriers,” and “stigma.” Keywords were combined with MeSH terms and truncated as appropriate and specific for each database. For this study, treatment uptake relates to at least one occasion of an outpatient, primary care, tele-health, face-to-face, pharmacological, online, harm reductive, or abstinence intervention. Six databases: Medline, CINAHL, Scopus, PsycINFO, Embase and the Cochrane library was searched. Citation chaining was used to include relevant articles in the screening process. Peer-reviewed articles containing qualitative and quantitative data were included. Limits were applied to studies from high-income countries only, English language (or translated into English) only, and a year restriction from 2000 to 2024 to ensure a comprehensive search that included recent studies. This review focuses on adult women (cis-gendered females aged 18 years and over). The search included women and alcohol use only, mixed substance use studies were included only if alcohol use data could be disaggregated. Studies including both men and women were only included if the results relating to women could be disaggregated.

Studies were excluded based on the following criteria:

1. Studies involving participants under 18 or over 80 years old.
2. Studies that did not present data specific to women. Mixed-gender studies were excluded unless they reported gender-specific analyses related to treatment-seeking behavior.
3. Articles not published in English.
4. Studies not conducted in high-income countries.
5. Studies focusing on minority groups of women, based on ethnicity, pregnant, or breastfeeding women, and women with co-occurring mental health challenges.
6. Studies investigating multiple substances where data on alcohol use could not be isolated.

**Figure 1.**
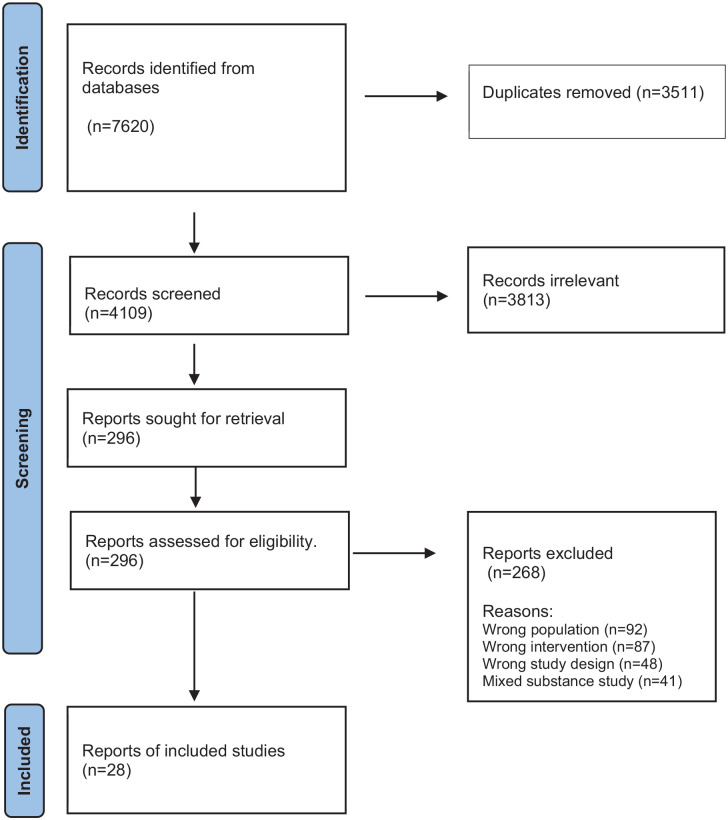
PRISMA flow diagram.

Although acknowledging gender diversity, the authors used “women” and “female” interchangeably, to refer to cisgender women. This approach was chosen to align with the study’s focus and existing research studies focused on cisgender women. The review was reported in accordance with the PRISMA flowchart and statement.^51^ Two reviewers checked all full-text articles and completed a table for excluded articles with reasons. After duplicates were removed, 4109 citations were retrieved from the database search. The initial title and abstract review indicated 122 potentially relevant articles. Further screening excluded 97 articles due to study outcomes not relating to access factors, and study populations that do not include data on women or alcohol use specifically. Twenty-five studies met the eligibility criteria and were included in the review (see Figure 1).

### Study screening and quality appraisal

The retrieved publications were imported into the Covidence software platform for screening. The authors systematically applied the inclusion criteria to assess eligibility for all identified studies. The first author (MA) undertook a quality appraisal of included studies using the Critical Appraisal Skills Program^52^ to assess quantitative and qualitative studies, the Mixed Methods Appraisal Tool version 2018^53^ for mixed-methods studies, and the cross-sectional 20 for cross-sectional studies.^54^ One other author (AD or JR) checked the appraisal, and any conflicts in appraisal were resolved through discussion with the review team. Only articles that scored as high quality (*n* = 25) were included. Low-quality articles were excluded due to a lack of clear research question inconsistencies in data interpretation and lack of consideration of risk bias.

### Data extraction and analysis

Data including study design, sample size, population characteristics, intervention details, and results were extracted and collated by one author (MA). The findings were summarized in tabular form according to the three key areas of the individual, societal, and organizational social ecological model and independently reviewed by other authors (AD and JR). A narrative synthesis, aligned with Popay et al. Guidance on narrative synthesis in systematic reviews was applied.^55^ The synthesis was driven by the theory that women’s treatment-seeking behaviors for AUD are shaped by factors across these levels.

Study results were coded and categorized as a basis for the narrative synthesis, reflecting Popay’s structured approach.^55^ Each study was reviewed to identify key elements contributing to treatment-seeking, and results were categorized based on intervention type and reported outcomes. Findings were tabulated to identify common characteristics, and thematic analysis examined emerging related to study outcomes. For instance, stigma emerged as a common societal barrier, whereas support networks facilitated access to care. Outcomes were categorized by whether the intervention increased women’s participation in treatment programs.

## Results

Twenty-five studies were included in the review. Four studies were conducted in Australia,^56–59^ 16 studies in the United States,^11,60–74^ 2 studies were conducted in Sweden,^75,76^ and 1 study each from the United Kingdom,^77^ France,^78^ and Belgium,^79^ were also included.

Seventeen studies were conducted in outpatient and community settings,^11,56–58,62–67,71,72,74–76,78^ three studies were conducted in criminal justice settings,^60,68,69^ and two in hospital settings.^59,77^ Two studies were conducted in both inpatient and outpatient settings,^73,79^ and one did not specify study locations.^70^

Of the studies included, 8 focused on non-treatment-seeking participants,^60,63,68,69^ and 10 studies focused on treatment-seeking participants.^58,59,62,65,67,72,75–78^. Five studies did not specifically distinguish participant types,^56,57,64,66,70^ though two included cohorts of both treatment-seeking and non-treatment-seeking individuals.^11,71^ This review included a mix of quantitative and qualitative studies with varying sample sizes and populations (see Table 1).

**Table 1.** Summary of study characteristics and findings.

Reference	Country	Facilitating factor	Study design	Study population	Aim	Findings
Amos et al., 2022^56^	Australia, all states	Individual; Problem recognition Organizational- use of non-heterosexist and inclusive language and directly asking about alcohol use in a culturally informed way	Cross-sectional survey study, multi-variate analysis.	Sexual minority women *n* = 2647, median age 31 years of age 60.50% reported risky use of alcohol and 15% were currently smoking tobacco.	To examine patterns of use and service engagement in sexual minority women who consume alcohol and tobacco.	Potential risky drinking was associated with living in outer urban or rural areas and being Australian born. Less than 3% of the sample has sought help for alcohol use. Seeking support was more likely as women aged, and with potentially risky drinking, and much more likely with self-perceived struggles with alcohol. Age was the only sociodemographic factor associated with seeking support for alcohol consumption in the past 12 months. Rates of recently seeking support were low (<3%) and increased with age, with those aged over 55 years old almost six times more likely to have sought support for alcohol consumption in the past 12 months compared to participants aged 18– 24 years.
Bernard et al.^57^	Australia, all states except Tasmania, 71% in urban areas	Organizational: digital health	Qualitative	*N* = 35 people, 46% female 19–58 years of age. Female average age 38.	To explore experiences of a multiple-session outbound telephone delivered psychological intervention for mild to moderate AUD.	Women reported valuing the convenience, anonymity, and collaborative approaches of the intervention; however, for some, the telephone-only format hindered accountability.
Begun et al.^60^	USA Wisconsin	Organizational: screening and brief intervention	Randomized control study using multivariate analyses and hierarchical regression analyses.	790 women were recruited during their incarceration in either Milwaukee County’s CJF or the House of Correction 276 women completed the intervention (59% of those eligible). Of these, *n* = 729 (92%) consented to randomization to either the intervention or treatment as usual group. 468 (64%) women were randomized into the intervention group and 261 (36%) were randomized into the treatment as usual (TAU) group. Primarily due to early release before the intervention could be delivered only 276 women completed the intervention (59% of those eligible).	To examine the impact of a prison in-reach brief screening and motivational interview on women’s alcohol and other substance use and treatment engagement during the first 2 months of community re-entry following release from prison.	The intervention and control group had no difference in treatment engagement. Yet, one half of the women at follow-up (47%) sought treatment in some type of program such as residential rehabilitation, counselling or attended a mutual-aid group after their release.
Black et al.^58^	Australia, all states	Societal: Social support	Qualitative, thematic analysis	*n* = 24 participants, 12 women (50%)	To inform website recruitment and retention strategies by exploring users’ motivations and experiences in using a novel, Internet intervention, the HSM program.	Evidence-based behavior change techniques, including social support, normative strategies, goal setting, and self-monitoring, were appealing to all participants, including those already making alcohol use changes and incidental users who joined through curiosity and desire for a challenge.
Bourdon et al.^61^	USA	Social: generational, genetic	Regression analysis	Relatives of probands who participated in the collaborative study on the Genetics of Alcoholism *n* = 4405 participants; *n* = 2026 female (46%)	(i) To describe service use for alcohol problems. (ii) To assess generational differences (silent [b. 1928 to 1945], boomer [b. 1946 to 1964], generation X [b. 1965to 1980], millennial [b. 1981 to 1996]) in help-seeking and treatment utilization, (iii) To examine sex differences across generations.	The study identified generational and sex differences in service utilization for AUD. Females were less likely than males to seek treatment, with notable age-related patterns. Distinct trends emerged in the interactions between sex and generation. Silent generation females were more likely to seek help compared to baby boomer females.
Bourion-Bedes et al.^78^	France, rural population	Individual: Trauma history and problem perception	Cross-sectional study, Multivariable logistic regression	Women from rural location, *n* = 50. with main diagnosis Axis 1 of the DSM-IV receiving outpatient treatment for alcohol dependence at a treatment program offered by the Healthcare Centre	To evaluate the association between sociodemographic and clinical characteristics and the probability of rural women with alcohol dependence seeking help on their own rather than being referred at a specialty treatment service.	Among rural women with problematic alcohol use the probability of self-initiated help-seeking at a specialist treatment service was greater for participants with no complementary health insurance. A history of physical and/or sexual trauma was associated with a significantly higher likelihood that participants would self-initiate help-seeking. compared to women who were referred by a clinician.
Calkins and Brock^62^	USA, Low-income community in Nebraska	Societal: role of intimate partner support	Systematic mixed methods descriptive study	Outpatients at a free clinic *n* = 59 (42 women) in an intimate-partner relationship lasting at least 6 months.	To systematically investigate the role of intimate partner support in alcohol use and to examine whether partner support serves a maladaptive function among individuals with a history of alcohol dependence.	Some supportive behaviors intended to be helpful might paradoxically lead to a greater probability of alcohol use leading to enabling of drinking and avoidance of consequences.
Conner et al.^63^	USA, 17 counties in western and central New York	Individual: Intra-personal consequences	Phase-II RCT Cross-sectional design.	Community sample of treatment naïve people, *n* = 349, *n* = 168 females (48%)	1. To test the efficacy of a one-session telephone intervention, CBT-TS, to promote the use of alcohol-related treatment and improve drinking outcomes in individuals whose AUD is severe but untreated. 2. To examine associates of alcohol-related intention to seek treatment in adults with severe untreated AUD recruited from the community	Alcohol-related consequences, rather than consumption levels, affects intention to seek treatment and these consequences differ by gender. Intrapersonal consequences – such as feelings of unhappiness, shame, and guilt – are linked with intention to seek treatment in women whereas financial consequences were more aligned with male intention.
Eubanks-Fleming^64^	USA; nationwide	Social: partner support and peer drinking levels	Hierarchical multiple regression analysis	133 individuals (50% female, 48% male), online survey. Median age 38 years.	To investigate the potential social influences that are associated with alcohol-related help-seeking, including perceived partner support, descriptive and injunctive subjective norms, and marital satisfaction.	Women’s help-seeking behavior remains generally consistent regardless of their partner’s acceptance or disapproval of their drinking. In contrast, men are less likely to seek help when faced with partner disapproval and more likely to seek help when their partner is supportive or permissive.
Finfgeld-Connett and Madsen^65^	USA; Missouri rural area	Organizational: affordability, accessibility	Randomized pilot investigation	*n* = 46 women, median age 50 years, ethnicity 100% white.	To evaluate the effectiveness of a Web-based, self-guided treatment program for women with problem drinking habits.	Web-based treatment for middle-aged women in rural areas is as effective in reducing problem drinking as a more traditional treatment approach. Web-based treatment options have the potential to move individuals with mild-to-moderate drinking problems from the preliminary stages of change to the action stage.
Francia et al.^59^	Australia; regional Victoria	Organizational: peer workers	Qualitative research article	20 participants, *n* = 14 female, *n* = 6 male, median age 42	To generate preliminary qualitative data to enable a better understanding of what aspects of the peer worker role positively influenced therapeutic alliances in the hospital setting.	Lived experience expertise strengthens therapeutic alliances between client and peer worker clinicians through established factors of trust, credibility, and hope due to the peer worker’s ability to approach informally and establish rapport through shared experience.
Gilbert et al.^66^	USA	Individual: problem perception	Quantitative, longitudinal study	People meeting DSM IV criteria for alcohol dependence, *n* = 2592 65% male, 35% female (*n* = 907).	1. To quantify gender differences in alcohol services utilization. 2. To explore potential gender differences in perceived need for help and reasons for not seeking help.	Problem severity, measured by AUD symptom count, was a highly significant predictor of treatment uptake, each additional AUD symptom was associated with 42% higher odds of perceiving a need for alcohol services (aOR = 1.42; 95% CI 1.28, 1.58, *p* < 0.001). Compared to men, women had approximately half the odds of utilizing any type of alcohol service (aOR = 0.53; 95% CI 0.33, 0.86, *p* = 0.01), less than half the odds of using specialty services (aOR = 0.41; 95% CI 0.19, 0.87, *p* = 0.02), and approximately one-third the odds of using 12-step groups (aOR = 0.39; 95% CI 0.21, 0.71, *p* = 0.002).
Grosso et al.^67^	USA; New Jersey	Individual: problem perception, lack of control	Qualitative, using a deductive coding foundation. and iterative strategy	Participants, *n* = 180 women with AUDs participating in a NIAAA-funded outpatient alcohol treatment research program.	1. To examine factors that motivated women to seek outpatient alcohol treatment. 2. To explore association between reported motivators and pre-treatment drinking. 3. To explore the association between marital satisfaction and report of hypothetically related motivators for each construct at baseline. 4. To explore the association between reported motivators and drinking outcomes over time including baseline and 3-, 9-, and 15-months post-baseline. 5. To explore the association between reported motivators for seeking treatment and readiness to change drinking behavior.	Findings indicate that, feeling out of control of the progression of one’s drinking may be particularly motivating for moving toward behavior change as compared to interpersonal–family and health concerns. Women who had health concerns at baseline had the highest contemplation stage scores. Women who had interpersonal–family concerns at baseline had the highest preparation stage scores, and women who had lack of control of AUD concerns at baseline had both the highest action and maintenance scores.
Hailemariam et al.^68^	USA; northeast	Individual: previous experience of treatment and trust in others	Quasi-experimental, cross-sectional data from ongoing intervention analysis.	Women in pretrial prison detention. *n* = 168	To explore correlates of engagement in AA.	The belief that others are trustworthy, older age and higher AUD severity were significantly associated with higher AA affiliation. AA affiliation was associated positively and significantly with age (*b* = 0.036, 95% CI 0.023, 0.050, *p* < 0.001), number of AUD criteria met (*b* = 0.071, 95% CI 0.006, 0.135, *p* = 0.033), and with general trust (*b* = 0.027, 95% CI 0.003, 0.051, *p* < 0.030).
Hemrage et al.^77^	United Kingdom, South London	Organizational: voucher incentive payment	Single-center, prospective, individually randomized pilot feasibility trial	30 people, 29% women *n* = 7 in comorbid AUD and ARLD in treatment specialist care setting	To explore the scope of voucher-based CM to promote engagement with integrated liver care	Overall positive views. Women cited motivational and behavioral routine benefits, financial incentive, and support source. A powerful tool for maintaining engagement.
Jakobsson et al.^75^	Sweden, urban location	Organizational: respectful, person-centered language	Qualitative content analyses using gender as a sorting factor.	12 people with AUD at specialist clinics in Swedish city (*n* = 5 women)	1. To explore the inducements for treatment-seeking for alcohol problems in women and men. 2. To identify what promoted or hindered treatment-seeking in women and men, and to what extent femininity and masculinity was reflected in the context of treatment-seeking	Shame and guilt created hindrances to treatment-seeking in women who did not want to be perceived as “not good enough,” and this made them avoid talking openly about their alcohol problems. Women found that comments about their drinking were humiliating and therefore ignored. Persons who showed both respect and authority – nurses, physicians, social workers – were able to put pressure on the women without being rejected.
Jirwe et al.^76^	Sweden	Individual: health, loss of control, restrictions to daily activities Organizational: preference for specialist care (GP shame)	Descriptive qualitative	13 participants, 5 women aged 65–78 seeking treatment.	To describe elderly people’s views on alcohol dependence, treatment-seeking, and treatment preferences	A Swedish study on women aged 65 to 78 found that stigma and shame were significant barriers to seeking treatment for alcohol dependence, particularly in primary care, where many feared judgements from their GPs and the potential for their alcohol use to overshadow other health concerns. Concerns for health and the life restrictions of alcohol use (driving) were of concern when managing feelings of isolation. More likely to seek treatment away from usual GP due to shame.
Johnson et al.^69^	USA	Social: trust and familiarity. Organizational Link to service	Feasibility study	Women in pre-trial detention, *n* = 14	To pilot test an enhanced referral approach introducing a 12-step volunteer who could accompany them to an AA meeting after release.	Providing linkage between women in jail and female AA volunteers who can accompany them to a meeting post release is feasible and acceptable. 80% of women attended one meeting with volunteer on release. 57% of women went to four or more. Participants had significantly fewer drinking days at follow-up 1 month after release.
Levine et al.^70^	USA	Organizational: primary care, SBIRT, gender-specific interventions	Literature review	27 studies	To examine recent literature on alcohol treatment access and engagement in women in the USA.	There are low rates of screening, brief treatment, treatment, and engagement in women in the USA. Further inquiry should focus on the impact of race/ethnicity on gender differences, improving provider and system policies to promote SBIRT and treatment engagement, and advancing digital interventions and implementation research to optimize the effectiveness of gender-responsive and culturally tailored interventions.
Room et al.^71^	USA: California	Social: family and peers	A probability sample of adult problem-drinking individuals using logistic regression analysis	*N* = 1590 (*n* ~ 926) and untreated (*n* ~ 672), 51% women (*n* = 580).	To predict having received pressure about drinking from a specific family member or friend	Family pressure, especially from children, was a factor in treatment uptake, with both younger and older women more likely to be encouraged by their children to seek treatment.
Rohn et al.^11^	USA	Individual: severity of use, psychological comorbidity and social: family history of dependence, genetic and environmental factors	Exploratory study	*N* = 719, 28% women *n* = 225 who comprised 21.6% of non-treatment-seekers and 30.4% of treatment	To analyze differences between treatment-seeking and non-treatment-seeking individuals with AD, including a focus on female participants.	Treatment-seeking people exhibit more severe alcohol dependence, higher levels of psychological distress, trauma-related symptoms, and a stronger family history of alcohol dependence compared to non-treatment-seeking people. A notable gender-based difference in treatment-seeking behavior emerged in this study, with women representing a higher proportion of treatment seekers (30.6%) compared to their representation among non-treatment seekers (22%).
Schamp et al.^79^	Belgium Flemish and the Walloon areas	Individual: Parental authority, problem recognition, knowledge of treatment. Organizational: knowledge of clinician	Qualitative purposive sampling	Females seeking treatment in outpatient and residential settings, *n* = 60. AUD *n* = 19 (30%)	To identify and obtain a better understanding of the barriers and facilitators for seeking treatment as experienced by substance using women.	Individual, societal, and organizational factors that facilitate treatment participation for women are interconnected and serve in differing ways. Individual factors include retaining or regaining child custody, intra-personal factors of shame and guilt, problem awareness, and hope for future. Knowledge of healthcare clinician and appropriate treatment intervention are organizational facilitators.
Small et al.^72^	USA, Alabama, Arkansas, Georgia, Louisiana, Mississippi, and Tennessee	Organizational: access and affordability	Descriptive quantitative, bivariate analyses	Participants, *n* = 733, *n* = 239 women (30%)	To describe the barriers/facilitators and need for treatment among a community sample of rural and urban women at-risk drinkers.	Women in this study identified two major barriers: treatment affordability and availability. Level of education, amount of daily drinking, age, income, access to private insurance, as well individual perceptions of cost related to seeking residential treatment, and number of wait days to see a mental health or physician for alcohol problems are all associated with gender and geography; many of these variables have been shown to be predictors of treatment entry.
Sugarman et al.^73^	USA	Organizational: digital intervention	Pilot interventional feasibility study	Women *n* = 60 inpatient detox and hospitalization and community settings, 18–74 years of age, 41% in first treatment episode.	To develop a web-based, gender-specific intervention as an addition to treatment as usual for women with (SUD).	97% finding the content helpful and a mean (CSQ) score of 34.8/40; alcohol was the primary substance for 56%, 41% were in their first treatment episode, while only 13% had attended women-only SUD programs, 58% had participated in women-only self-help groups.
Wu and Ringwalt^74^	USA	Social: less family support for treatment. Organizational: primary care	Data were drawn from the 1999 National Household Survey on Drug Abuse.	*N* = 32,628 adults aged 18–64 years. Women *n* = 17,379 (53%), men *n* = 15,249 (47%).	To examine the extent and characteristics of alcohol dependence and the perceived need for and use of alcohol treatment services among women compared with men in a non-clinical sample of adults 18–64 years of age.	Women with alcohol problems are less likely than men to receive support from family or friends to seek treatment and are more likely to seek help in non-addiction treatment settings.

CJF: Criminal Justice Facility; HSM: Hello Sunday Morning; RCT: randomized controlled trial; CBT-TS: Cognitive Behavioral Therapy for Treatment Seeking; 95% CI: 95% confidence interval; aOR: adjusted odds ratio; AUD: alcohol use disorder; NIAAA: National Institute on Alcohol Abuse and Alcoholism; AD: alcohol dependence; SBIRT: screening, brief intervention and referral to treatment; AA: alcoholics anonymous; GP: general practitioner; SUD: substance use disorder; CSQ: client satisfaction questionnaire.

Two studies identified several key differences between treatment-seeking and non-treatment-seeking women. Treatment-seekers in a study that comprised of 28.4% females^11^ exhibited higher average daily alcohol consumption (13.6 drinks per drinking day, SD = 7) compared to non-treatment-seekers (8.6 drinks per drinking day, SD = 5.6), higher levels of psychological distress (depression and anxiety), greater trauma-related symptoms (early-life stress events and physical neglect), and a strong family history of alcohol dependence. These factors, alongside perceived illness severity and genetic or environmental influences, were found to significantly motivate help-seeking behavior.

Furthermore, treatment seekers were more likely to report unsuccessful attempts to reduce drinking, spending considerable time drinking, missing activities, experiencing psychological issues, and encountering withdrawal symptoms compared to non-treatment seekers.^11^ Family pressure, especially from children, was a significant factor in treatment uptake, with both younger and older women appeared to be more likely to be encouraged by their children to seek treatment.^71^

### Individual factors

The severity of alcohol problems, as measured by the Alcohol Use Disorders Identification Test (AUDIT), is a strong predictor of treatment uptake. Gilbert et al.^66^, analyzing data from NESARC waves 1 and 2, found that each additional symptom of AUD increased the likelihood of perceiving a need for treatment by 42%. Similarly, Grosso et al.^67^ reported that 31% of participants cited concern about their increasing alcohol consumption as a key motivator for seeking treatment, highlighting the role of perceived severity. Additionally, two studies on incarcerated women in the United States revealed a strong link between higher AUDIT scores and engagement in treatment following release.^61^ A further two studies reported older age as a predictor of help-seeking.^56,61^

Family-related concerns were found to be key motivators. For instance, 38% of women seeking treatment in a study by Grosso et al. cited the impact of drinking on their spouse and children as a primary motivator.^67^ Retaining or regaining custody of children was also reported as a strong motivator in a Belgian study of residential and outpatient treatment-seeking women.^79^ Negative self-image, guilt, shame, and unhappiness were additional predictors of help-seeking behavior in women without prior treatment experience.^63,79^ Other motivators included aesthetic and lifestyle concerns, such as wrinkles, weight gain, “drunk dialing,” and cognitive impacts like blackouts.^75^

Using data from individuals with alcohol-related issues who participated in the Collaborative Study on the Genetics of Alcoholism in the United States, Bourdon et al.^61^ examined generational differences in treatment-seeking behavior. Their study found that women in their 20s are less likely than men to seek help, whereas women in their teens, 30s, and 40s have similar treatment-seeking rates to men. Interestingly, women over 50 years are more likely than men to engage in treatment. This trend is also evident in the Silent Generation (born 1928–1945), where women seek treatment more often than their Baby Boomer counterparts, a pattern not observed among men. Among elderly women, the inability to control alcohol use and restrictions on activities like driving were key motivators for seeking treatment, according to a Swedish study.^76^

#### Co-occurring mental health challenges

Women with AUD in the United States are more likely than men to have co-occurring mental health disorders (39% versus 24%) and more likely to experience mental health challenges related to a history of physical or sexual abuse (48.5% of women versus 2% of men).^66,72^ A French study found women with AUD were twice as likely to have attempted suicide compared to men (43% versus 23%), were more likely seek treatment for co-occurring depression, and to drink to cope with distressing emotions.^63,78^ Similarly, Rohn et al. found that women often seek help for alcohol use in mental health settings, attributing their alcohol use to depression and anxiety while downplaying the severity of their alcohol consumption.^11^ Small et al. study also identifies several barriers for women in access to mental healthcare compared to men. Women report significant barriers related to the affordability and availability of mental health treatment compared to men, with a higher proportion of men having insurance coverage, with women more likely to view the cost of seeing a mental health provider, including transportation and childcare expenses, as prohibitive.^72^

### Societal factors

Findings indicate that partner and child support or disapproval, and the drinking habits of close friends, can strongly influence treatment-seeking behavior. Indeed, women with AUD were more likely than men to have a family history of alcoholism.^72,76,78^ A study by Eubanks-Fleming^64^ found that women’s help-seeking behavior remained consistent regardless of their partner’s approval or disapproval of their drinking. In contrast, men were less likely than women to seek help when facing partner disapproval and more likely to do so when their partner was supportive or permissive.^65^ However, in some cases, partner support intended to help can paradoxically enable alcohol use by shielding a woman from its consequences and reducing motivation to change.^62,64^

### Organizational factors

Cost is a key factor that impacts women’s treatment-seeking for AUDs. In the United States, where healthcare is largely privatized and expensive, women’s perceptions of the affordability of alcohol treatment have been found to negatively affect their treatment motivation.^65^ Interestingly, Small et al.^72^ reported that a higher proportion of women than men (33.77% versus 23.67%) thought that the cost of talking to a primary care clinician or specialist about their alcohol use was more than they could afford, but they would consider residential treatment despite the cost. This factor may be understood considering the study’s other findings that women reported lower levels of social support compared to men and may reflect their perception of the supportive environment of residential treatment.^72^

Women were likely to access public services in countries with universal healthcare. For example, a study from France found that women without private health insurance were 5.1 times more likely to seek specialty treatment in public clinics without a primary clinician referral.^78^ This implies that women may face different challenges and opportunities in accessing alcohol treatment depending on the type and level of healthcare coverage they have. A novel U.K. study exploring voucher-based incentives for women with AUD and liver disease found that vouchers enhanced motivation for treatment, supported behavioral routines, and offered valued financial and emotional support. Although one participant remarked that it “feels like bribery,” overall satisfaction was high.^77^

#### Clinician knowledge

Women are more likely than men to seek help in non-substance use treatment settings,^74^ yet there are low rates of screening, brief intervention, and referral to treatment (SBIRT) in primary care settings.^70^ Levine et al. found that alcohol screening occurred in only 2.6% of 19,213 visits, with no overall gender difference, though older women were less likely than men to be asked about drinking (6.8% versus 9.8%) and to receive treatment information (0.7% versus 2%).^70^ Similarly, some women, particularly older ones, report delays in getting help due to limited information or lack of referral from their GP in a Belgian study.^79^ However, Jirwe et al. found that women aged 65–78 often avoided seeking help for AUD from their GPs due the fear of judgment, instead preferring specialist services that offered greater privacy and expertise.^76^ Moreover, clinicians who demonstrated respect and knowledge of treatment options were found to be more effective than friends or family at motivating women to access alcohol treatment in a study by Jakobsson et al.^75^

#### Digital and telehealth interventions

Barriers of affordability and accessibility are often greater for women than men.^65^ Participants in a study from Australia reported satisfaction with online sites that provide harm reduction strategies in a convenient, free, anonymous way.^59^ Similarly, Bernard et al. highlighted that telephone interventions offer accessibility, convenience – crucial for women balancing paid work and other responsibilities – and satisfaction with harm reduction strategies and collaborative treatment planning.^57^

Treatment barriers related to affordability and accessibility are often higher for women than men.^72^ Web-based and telehealth treatment approaches may reduce barriers. A study of women living in rural United States reported high rates of acceptability of web-based interventions,^65^ and high rates of satisfaction with web-based interventions were reported by Sugarman et al. in a pilot feasibility study that proposed a web-based, gender-specific, intervention.^73^ Furthermore, an Australian study^57^ found participants were satisfied with online harm reduction strategies that are convenient, free, and anonymous, and Bernard et al. noted that telephone interventions diminished accessibility barriers for women balancing work and other priorities when contemplating alcohol treatment.^57^

#### Healthcare providers with lived experience

Evidence from three studies shows that when female volunteers from alcoholics anonymous (AA)– a peer-led mutual support program that promotes abstinence – connected with incarcerated women with AUD, these participants were more likely to attend AA meetings after release.^60,68,69^ This peer support model parallels findings from hospital settings, where an Australian study found AOD peer workers in consultation-liaison services effectively facilitated treatment engagement through authentic, informal relationships built on mutual understanding of lived experience.^59^

## Discussion

Across the 25 included studies, we found examples of individual, societal, and organizational factors in more than 5 high-income countries that influence women’s treatment uptake. The findings highlight the complex interplay of factors that hinder women’s uptake of AUD treatment including low perception of need, high levels of shame, co-occurring mental health challenges, and financial disparities compared to men.

### Problem perception

Similar to the broader literature this study found that women’s motivators to change their alcohol use – such as, personal beliefs, social norms, severity of alcohol use symptoms, wanting to repair relationships, or to regain or retain custody of children – strengthened problem perception which is strongly linked to treatment uptake.^67^ There is mixed evidence on whether older or younger women, or those with higher or lower levels of education and employment, were more likely to acknowledge their alcohol use problems.^36,37,80^ Though, reaching a turning point or crisis, such as realizing the unsustainability of their alcohol use and the negative impacts of their drinking upon their self-esteem and family, was found to be a dominant factor in problem recognition.

Women who consume alcohol often experience harsher criticism in society, which is arguably related to their likelihood to experience higher rates of internalized shame compared to men.^38^ Lower problem recognition can function as a coping response, allowing women to avoid internalized stigma and retain a positive drinking identity.^81^ However, shame and lack of problem recognition can lead to concealment of drinking, denial, and shunning of treatment.^79^ Even when problem recognition is present, factors such as affordable, accessible, gender-responsive services can maintain barriers to treatment uptake, financial support, and childcare availability play a critical role in enabling treatment uptake.^33,82^

### Social environments

Positive peer and family support, as well as the absence of a drinking partner, play an important role in facilitating women to seek help for AUD. The term support in this context is complex and may be better viewed through the lens of support adequacy, where the quality and quantity of help meet the woman’s needs.^62^ The effectiveness of programs such as the Community Reinforcement and Family Training (CRAFT) reflects this concept by equipping parents, spouses, and other relatives, with behavioral strategies to encourage treatment-seeking in individuals refusing help.^83^ Similar approaches can be particularly relevant for women with AUD, who often report less emotional support for change compared to men.^74^

Kippax’s theory of social change that posits community is central to the process of transforming social norms and practices around alcohol consumption. Arguably, wellness and sober-curiosity movement, and the popularity of sober months, have the potential to increase the social acceptability of alcohol abstinence periods for women, and to promote connection to peer networks.^84^ Temporary abstinence campaigns (TACs) such as “Dry January,” “FebFast,” and “Ocsober,” not only encourage abstaining from alcohol for a month, but also connection to peers and fundraising for a cause. However, research indicates that TACs primarily target and attract motivated social drinkers rather and could potentially put dependent drinkers at risk by reducing the likelihood of them seeking professional treatment.^85,86^ There is also a need for further research to evaluate the potential effects of these campaigns for women.^87^

Peer and mutual-aid support groups, such as AA and self-management and recovery training, are strategies that facilitate a social practice approach that also includes gender-specific formats. Women-only groups are available and are based on the principle of sharing experiences and providing social and practical support among others who have lived through similar challenges and can help reduce the isolation that women with AUD often face.^88^ Other women-specific mutual aid groups such as Women for Sobriety in the United States, reflect varied approaches that women can choose from to align with their values, beliefs, and recovery goals. Furthermore, online mutual aid networks for alcohol use demonstrate higher female participation and satisfaction rates compared to males, likely due to the flexibility they offer, and accommodating commitments that may prevent access to formal treatment.^89^

### Knowledge of treatment services

Women’s knowledge of treatment services, as well as their previous experiences with treatment providers, can affect their treatment-seeking behavior.^80^ Awareness of harm reduction and person-centered care is important for women who fear abstinence-only approaches, as these methods may not suit all women.^8^ As women are more likely than men to drink to cope with negative emotions and stress, they are also more likely to seek help for alcohol-related depression and anxiety, rather than AUD, via mental health or primary care settings.^37,63^ Therefore, knowledge of AUD treatment is crucial for primary clinicians, who should be aware of the role that alcohol plays in women’s lives and the shame they may feel about seeking help.^10,90^

Primary care settings are a key entry point to treatment for women and SBIRT for AUD delivered by primary health providers has been shown to increase the odds of treatment uptake by 4.7 times compared to those who did not receive a SBIRT intervention.^91^ However, evidence of its effectiveness is mixed. One study found women responded better to stepped-care interventions,^92^ and Frost et al.^93^ reported that patients receiving documented brief interventions were less likely to access effective treatments or AUD medications, particularly those with a prior-year AUD diagnosis. These findings emphasize the critical role of the “Referral to Treatment” component in the SBIRT model, ensuring that patients transition effectively from brief interventions to appropriate care.^94^

Moreover, a narrative review by Clarke et al.^46^ observed that women over 50 were 25% less likely than men to be asked about their alcohol use by their general practitioner (GP) and received less alcohol advice than their male counterparts. The same study found that while women over 50 are likely to downplay their alcohol use when discussing with GPs, over half of the respondents expressed interest in information on alcohol medication. This suggests that there is a gap between the demand and supply of pharmacological interventions for women that can be addressed in primary care services such as nurse-led alcohol clinics, where evidence-based pharmacological and harm reduction interventions can be provided in accessible and discreet community health settings.^95^

The COVID-19 pandemic has expanded treatment options for women with AUD through digital therapeutics, telehealth, online peer support, and anonymous web-based applications, reducing the cost, time constraints, and stigma of seeking treatment.^96,97^ A systematic review by Hai et al.^98^ highlighted the effectiveness of technology-based interventions for women, with studies by Tait et al. and Simpson et al. indicating a higher representation of women in online trials.^43,99^ Women are more likely than men to seek health information online,^100^ and while both genders benefit similarly from computer-assisted interventions, studies indicate that women report higher levels of acceptability than men.^101^ Findings suggest that digital interventions designed specifically for women may offer a private, non-judgmental environment and can be tailored with gender-specific content to address distinct needs such as trauma, caregiving responsibilities, and co-occurring mental health conditions.^73^

### Integrated care

Comorbid psychiatric disorders in women are more closely linked to gender rather than geographic factors, indicating that women have distinct needs that influence their treatment entry.^80^ Women are more likely than men to seek help for depression and anxiety, rather than AUD, via mental health or primary care settings.^37,63^ Distress from mood disorders and anxiety often co-occurs with and is worsened by AUD, significantly increasing the likelihood of seeking treatment.^102^ Data from two large U.S. surveys suggest that women with AUD may have a preference for seeking mental health treatment, whereas men are more likely to seek AUD-specific treatment.^103^ Furthermore, treatment-naïve individuals highlighted key factors influencing treatment access, such as integration of alcohol and other drug services within general medical settings.^104^ The data suggest that while alcohol use may be the underlying issue requiring treatment, women may be more likely to seek help through non-specialist services, underscoring the need for integrated care models.^47^

### Women-focused care

Evidence suggests that women-specific treatment can reduce barriers to healthcare access and improve treatment retention for women with AUD.^33^ A women-responsive model of care includes trauma-informed, strength-based, and resilience-oriented practices. It recognizes the differences in drinking patterns between women and men and how life transitions, such as child-rearing, menopause, and ageing, affect the gendering of drinking.^105^ This approach aligns with the Australian National Drug Strategy’s focus on evidence-based, accessible treatment, the SAMHSA guidelines in the United States, and the U.K. government’s emphasis on women-specific care for alcohol and mental health disorders.^106–108^

A body of literature has discussed the facilitators and barriers that women-focused treatment may provide though evidence can diverge. Studies found that women-focused treatment was more likely to increase service utilization and that women were less likely to access treatment when women-only services were not available.^10,82^ Though others argue that women-only approaches are not a universal remedy or preferred by women,^109^ whereas another opinion suggests that AUDs can influence decision-making and interpersonal relationships, potentially affecting women’s perceptions of the treatment they believe is necessary.^110^ However, services that offer women-sensitive treatment – feasible even in mixed-gender settings – are associated with increased treatment access for women.^38^

### Implications for practice, policy and future research

The findings of this review underscore the need to integrate gender-specific, personalized interventions tailored to women’s unique treatment needs, with a priority on developing women-focused AUD treatment programs that incorporate digital tools and resources. Future research should refine gender-sensitive interventions, assess long-term effectiveness, and explore applicability across diverse populations for broader inclusivity and impact.

### Limitations

This study has some potential limitations. First, while acknowledging gender diversity, it is not always possible to identify transwomen as they do identify as “female” and “women.” The authors’ interchangeable use of “women” and “female” reflects a research strategy of a cisgender lens while operating within established research conventions. Second, this study’s focus and inclusion criteria and search strategy limiters, resulted in the non-inclusion of some groups of women. First Nations and women with co-occurring substance use disorders and comorbid mental health diagnoses, pregnant and breastfeeding women, and transwomen were not represented in this systematic review. We recognize that these categories encompass a wide range of experiences and challenges, which are essential to consider in comprehensive research. The decision to exclude studies on subgroups of pregnant or breastfeeding women was determined based on the specific treatment needs of these women. Treatment of pregnant and breastfeeding women necessitates specialist knowledge about the impact of AUD treatment on pregnancy, birth, and lactation, and involvement of obstetrics and pediatrics due to potential impacts on infant health. It was determined that the treatment barriers for this cohort required a unique analysis that was beyond the scope for our study. Therefore, the findings may not reflect their specific needs and experiences.

Lastly, limitations arose from small sample sizes in some qualitative studies and an over-representation of educated, primarily Caucasian participants. Additionally, the predominance of U.S.-based studies limited the generalizability of findings.

## Conclusion

This review adds to the existing literature on women and alcohol use by examining the facilitators of alcohol treatment from a social ecological perspective. The findings of this systematic review suggest that facilitating factors of treatment uptake in women with AUD, include relational and personal goals, societal environments and normative expectations, and the knowledge of alcohol treatment from an individual and organizational perspective. Treatment options that are women-focused, flexible, and discreet are needed to increase treatment-seeking in this population group. Sober curiosity movements and alcohol-free months, and integration of digital treatment strategies reflected in post-COVID-19 healthcare trends, may influence women’s attitudes and motivations toward alcohol treatment.

## Supplemental Material

sj-docx-1-whe-10.1177_17455057251363713 – Supplemental material for Factors that facilitate treatment uptake for women with alcohol use disorders in high-income countries: A systematic review Supplemental material, sj-docx-1-whe-10.1177_17455057251363713 for Factors that facilitate treatment uptake for women with alcohol use disorders in high-income countries: A systematic review by Melise Ammit, Jo River, Mark Montebello and Angela Dawson in Women's Health
